# On the Action of Medicinal Preparations of Iron on the Teeth

**Published:** 1866-06

**Authors:** John Smith

**Affiliations:** Surgeon-Dentist to the Royal Infirmary


### Selections.

---

### ON THE ACTION OF MEDICINAL PREPARATIONS OF IRON ON THE TEETH.

By John Smith, M. D., F. R. C. S., Surgeon-Dentist to the Royal Infirmary.

The preparations of iron in medicinal use are very generally supposed to exert, in a direct manner, an injurious influence on the teeth of patients for whom they are prescribed. Complaints regarding their destructive tendency in this respect are familiar both to the general practitioner and to the dentist. With the view of ascertaining how far such complaints are well grounded, the experiments I have here briefly to notice were made.

*Eight* of the compounds of iron in most general use as remedial agents, and along with them one or two other non-ferruginous compounds, sometimes suspected of injuring the teeth, were selected, and in solutions of these compounds the same *number* and the same *kinds* of human teeth were immersed. In each of the separate solutions four teeth were placed; and in all cases these four teeth consisted of a sound canine and bicuspid, and a decayed upper and lower molar. The solutions consisted of:

I. Sach. carb. ferri, gr. xx.; aquæ, ℥ i.

II. Carb. ferri, gr. xx.; aque ℥ i.

III. Syrup. phos. ferri, ℥ ss.: phos. ferri, gr. x.; aquæ, ℥ ss.

IV. Syrop. iod. ferri, ℥ ss.; aquæ, ℥ ss.

V. Citrat. quiniæ et ferri, ij.; aquæ, ℥ i.

VI. Vinum ferri, ℥ ss.; aquæ, ℥ ss.

VII. Sulphat. ferri, ʒ ss.; aquæ, ℥ i.

VII. Tinct. mur. ferri, ʒ ij.; aquæ, ℥ i.

At the same time the following non-ferruginous solutions were tested;

I. Sulphate of quinine, gr. v.; sulphuric acid, gtt. i.; water, [℥ ss.

II. Phosphoric acid dilute, ℥ ss.; water, ℥ ss.

III. Condy's fluid (crimson,) ℥ ss.; water, ℥ ss.

On examining the respective solutions after twenty-four hours, the teeth were found unaltered in those of the carbonate and saccharine carbonate of iron, the phosphate of iron, the iodide of iron, the citrate of quinine and iron, and in that of the sulphate of quinine. In the solution of the vinum ferri, the liquid itself was somewhat turbid, the teeth, however, seeming to be untouched. In that of the muriate of iron, a turbid sediment filled the bottom of the bottle, and covered up the teeth from view; the fangs of the teeth were somewhat soft and flexible, and the enamel easily scraped down. The sediment, under the microscope, presented an amorphous granular appearance. In that of the phosphoric acid they seemed somewhat flexible at their more slender parts, such as the points of the fangs; and the enamel looked opaque and chalky, but did not feel crumbling or soft. In the solution of Condy's fluid they were deeply stained, but in no way altered in texture.

The teeth in the different solutions were allowed to remain ten days longer, and on examining them at the end of that period, neither those in the carbonate or saccharine carbonate of iron, the phosphate of iron, the iodide of iron, nor the citrate of iron and quinine, presented any further change, except that those in the saccharine carbonate were slightly blackened—the discoloration, however, being superficial, and nearly all removable by brushing. A ropy sediment adhered to those in the sulphate of quinine, and perhaps a very slight softening of the surface of the fang might be present. The ropy sediment presented, under the microscope, the appearance of a mass of acicular crystals of various sizes, interspersed with threads or fibres of some kinds.

In the solutions of the vinum ferri, the sulphate of iron, the muriate of iron, the phosphoric acid, and of Condy's fluid, certain changes were observable. In the vinum ferri the cloudy precipitate was somewhat increased, and the teeth were dark and discolored, especially in the fangs, but otherwise uninjured. In the sulphate of iron solution a very copious precipitate had formed. The teeth were not softened throughout their whole thickness, but had a layer of soft substance covering in what of the enamel and dentine remained hard beneath. They were also considerably discolored. In the solution of the muriate of iron, a large deposit of the cloudy precipitate had accumulated, and in this the teeth lay buried. Their fangs were very soft, and quite flexible, and the enamel was wasted away, and on being touched, crumbled down like chalk. In those again, immersed in the phosphoric acid solution the fangs were quite flexible, and were diminished in bulk; the enamel could be deeply scratched with any blunt instrument, and felt like Derbyshire spar. It was more wasted, but not so soft as that of the teeth in the muriate of iron solution. Nothwithstanding this loss of salts of lime, on drying by exposure to the air these teeth have again become hard and unyielding. In the Condy's solution, the teeth were covered by a very dark incrustation, which, however, could be nearly all removed by brushing, or still more effectually by applying dilute muriatic acid; otherwise they were uninjured. The solution itself had become almost colorless.

From these facts it would appear that certain preparations of iron, when directly applied, do exercise a powerful effect on the substance of the teeth. And the ratio of the effects obtained would seem to prove, that of all the preparations employed in these experiments that of the tincture of the muriate of iron acts most powerfully, the sulphate of iron next, and next to that again, although in comparison very immaterially, the vinum ferri—the other preparations of iron appearing to be inert.

Of the other substances experimented with, phosphoric acid seems the only one producing injurious effects on the teeth, which it does, however, to a very marked extent—. *Edinburgh Medical Journal.*

